# *Magnaporthe oryzae* Chloroplast Targeting Endo-β-1,4-Xylanase I *MoXYL1A* Regulates Conidiation, Appressorium Maturation and Virulence of the Rice Blast Fungus

**DOI:** 10.1186/s12284-022-00584-2

**Published:** 2022-08-12

**Authors:** Ammarah Shabbir, Wajjiha Batool, Dan Yu, Lili Lin, Qiuli An, Chen Xiaomin, Hengyuan Guo, Shuangshuang Yuan, Sekete Malota, Zonghua Wang, Justice Norvienyeku

**Affiliations:** 1 Key Laboratory of Green Prevention and Control of Tropical Plant Diseases and Pests, Ministry of Education, College of Plant Protection, Hainan University, Haikou, China; 2 Hainan Yazhou Bay Seed Laboratory, Sanya Nanfan Research Institute of Hainan University, Sanya, China; 3 State Key Laboratory for Ecological Pest Control of Fujian and Taiwan Crops, College of Life Science, Fujian Agriculture and Forestry University, Fuzhou, 350002 China; 4 Institute of Oceanography, Minjiang University, Fuzhou, 350108 China; 5 College of Plant Protection, Shenyang Agriculture University, Shenyang, China

**Keywords:** Xylanases, *Magnaporthe oryzae*, Chloroplast targeting peptide, Pathogenesis, Rice blast disease

## Abstract

Endo-β-1,4-Xylanases are a group of extracellular enzymes that catalyze the hydrolysis of xylan, a principal constituent of the plant primary cell wall. The contribution of Endo-β-1,4-Xylanase I to both physiology and pathogenesis of the rice blast fungus *M. oryzae* is unknown. Here, we characterized the biological function of two endoxylanase I (*MoXYL1A* and *MoXYL1B*) genes in the development of *M. oryzae* using targeted gene deletion, biochemical analysis, and fluorescence microscopy. Phenotypic analysis of ∆*Moxyl1A* strains showed that *MoXYL1A* is required for the full virulence of *M. oryzae* but is dispensable for the vegetative growth of the rice blast fungus. *MoXYL1B*, in contrast, did not have a clear role in the infectious cycle but has a critical function in asexual reproduction of the fungus. The double deletion mutant was severely impaired in pathogenicity and virulence as well as asexual development. We found that *MoXYL1A* deletion compromised appressorium morphogenesis and function, leading to failure to penetrate host cells. Fluorescently tagged MoXYL1A and MoXYL1B displayed cytoplasmic localization in *M. oryzae*, while analysis of MoXYL1A-GFP and MoXYL1B-GFP *in-planta* revealed translocation and accumulation of these effector proteins into host cells. Meanwhile, sequence feature analysis showed that MoXYL1A possesses a transient chloroplast targeting signal peptide, and results from an Agrobacterium infiltration assay confirmed co-localization of MoXYL1A-GFP with ChCPN10C-RFP in the chloroplasts of host cells. MoXYL1B, accumulated to the cytoplasm of the host. Taken together, we conclude that *MoXYL1A* is a secreted effector protein that likely promotes the virulence of *M. oryzae* by interfering in the proper functioning of the host chloroplast, while the related xylanase MoXYL1B does not have a major role in virulence of *M. oryzae*.

**Supplementary Information:**

The online version contains supplementary material available at 10.1186/s12284-022-00584-2.

## Background

The plant cell wall, composed of celluloses, hemicelluloses, and pectin, is the first obstacle a pathogen encounters in plant-pathogen interaction (Kubicek et al. 2014). For this reason, pathogens produce and secrete an array of plant cell wall degrading enzymes to weaken and overcome this initial barrier (Brito et al. 2006; Kubicek et al. 2014; Mori et al. 2008; Win et al. 2012). Cell wall degrading enzymes (CWDEs) are key virulence factors for pathogens as they help not only in host cell invasion but also facilitate the depolymerization of plant macromolecules to small molecules that can be acquired as nutrient resources by the pathogen (Fernandez et al. 2014). CWDEs can also act as elicitors of the plant defense response (Ramonell et al. 2002; Ryan and Farmer 1991) CWDEs play vital roles in promoting the successful invasion and colonization of host tissues by phytopathogenic fungi during early and late stages of pathogen-host interaction(Gibson et al. 2011). The first CWDE found to be necessary for virulence was pectate lyase in *Erwinia chrysanthemi* (Roeder and Collmer 1985), followed by endo polygalacturonase in *Aspergillus flavus* (Shieh et al. 1997) and ethylene inducing xylanase in *Trichoderma spp.* (Beliën et al. 2006).

Xylan is the principal polysaccharide component of hemicellulose, which, with cellulose and lignin, makes up the majority of plant cell wall biomass, including that of plants of the *Gramineae* family (Collins et al. 2005). It consists of a 1,4-linked D-Xylp backbone with side branches of AraF and GlcpA (Scheller and Ulvskov 2010). Glycoside hydrolases (GHs) are the broad category of enzymes capable of breaking glycosidic bonds in oligosaccharides and polysaccharides. Most fungal xylanases belong to the GH10 family of high molecular mass endoxylanases (> 30 kDa) and the GH11 family of lower molecular mass endoxylanases (< 30 kDa) (Biely et al. 1997; Lagaert et al. 2009).

Endo-1,4-β-xylanases (EC 3.2.1.8) cleave β-1,4-linkages between xylose units and play a significant role in fungal penetration and colonization (Beliën et al. 2006; Dornez et al. 2010), (Walton 1994) and induce necrosis in host tissues The xylanase encoding genes in *C. carbonum* (Apel-Birkhold and Walton 1996), *F. oxysporum* (Gómez-Gómez et al. 2002) and *F. graminearum* (Sella et al. 2013) plays non-essential role in virulence*.* However, endo-β-1,4-xylanase encoding genes in other plant pathogens have been shown to have key roles in virulence, including *xynB* in *Xanthomonas oryzae pv. Oryzae* (Rajeshwari et al. 2005), *xyn11A* in *Botrytis cinerea* (Brito et al. 2006), SsXyl1 in *Sclerotinia sclerotiorum* (Yu et al. 2016) and VmXyl1 in *Valsa mali* (Yu et al. 2018). Two xylanases belonging to the GH10 family, *ppxyn1* and *ppxyn2* were sufficient to impart virulence to an oomycete *Phytophthora parasitica* for infection of tomato and tobacco plants (Lai and Liou 2018).

The blast fungus *Magnaporthe oryzae* (*syn Pyricularia oryzae*) is a hemibiotrophic filamentous ascomycete threatening worldwide rice and wheat production (Dean et al. 2012; Ebbole 2007). The life cycle of the fungus starts with a three-celled conidium adhering to the leaf surface and undergoing various morphological changes to form a dome-shaped appressorium (Talbot 2003). A turgor pressure of 8 MPa is built up inside the appressorium that is translated into a mechanical force used to form a penetration peg to breach the leaf cuticle and enter the host (Raman et al. 2013; Wang et al. 2011). The genome of *M. oryzae* contains 20 xylanase genes that encode six glycoside hydrolases in the GH10 family, five in the GH11 family, and nine in the GH43 family (Dean et al. 2005). This prevalence of xylanase genes suggests that they may serve important roles in the life cycle of the blast fungus. In a previous study, Endo-β-1,4-Xylanases in *Magnaporthe oryzae* were silenced to reveal their potential roles in fungal virulence (Nguyen et al. 2011). In this study, the authors characterized xylanases that were specifically upregulated during wheat infection and reported *MGG_07955* (GH11) and *MGG_08424* (GH11), referred to as MoXYL1A and MoXYL1B, to be non-expressed xylanases. We hypothesized that these two xylanases likely play roles that are directly related to the pathogenicity, or virulence of the rice blast fungus. We therefore investigated the contribution of these two Endo-β-1,4-Xylanases in the virulence of *M. oryzae* as well as their intrinsic function as secreted effector proteins.

## Materials and Methods

### Strains and Culture Conditions

*Magnaporthe oryzae* isolate, Guy11 protoplast, was used for generating gene deletion mutant strains for functional characterization of *MoXYL1.*

The strains were cultured on Complete Medium (CM; Yeast extract 6 g/L, Casamino acid 6 g/L, Sucrose 10 g/L, and Agar 20 g, dissolved in double distilled water), supplemented with antibiotic (streptomycin 100 µg/100 mL), under standard incubation conditions of 28 °C (Chen et al. 2008).

For sporulation assays, Rice Bran Media (RBM; Rice bran 40 g/L and agar 15 g, dissolved in dd water with pH adjusted to 6) (Zhang et al. 2019), Straw Decoction and Corn media (SDC; Rice straw 200 g, corn agar 40 g/L,15 g agar in 1 L double distilled water) (Chen et al. 2010) and CM-II medium (50 mL 20 × nitrate salts, 1 mL trace elements, 10 g glucose, 2 g peptone, 1 g yeast extract, 1 g casamino acids, 1 mL vitamin solution, 15 g agar in 1 L distilled water) (Chen 2018) were used. Culture plates were kept in dark conditions for 7-days, followed by scratching hyphae and exposing the plates for 3-days to fluorescent light at 28 °C (Aliyu et al. 2019; Zhang et al. 2017).

For generation of competent cells, the *Escherichia coli* strain DH5α was cultured on lysogeny broth (LB) medium (10 g tryptone, 5 g yeast extract, and 10 g NaCl in 800 mL dH_2_O), with pH adjusted to 7 by adding 1 M NaOH, before adding up more deionized water to make up 1 L with distilled water (Abdul et al. 2018).

### Microscopy Assays to Confirm Protein Secretion

MoXYL1 protein secretion was observed by inoculating strains expressing GFP fusion constructs on barley leaves (Chen 2018). Mycelial plugs were prepared by shaking small pieces of the strains in liquid CM in a 28 °C shaking incubator at 120 rpm for 3-days. Barley leaves placed on moistened filter papers were inoculated with the mycelial plugs. The inoculated leaves were incubated under dark conditions at 28 °C. The leaf sheath was excised with a sterilized fork and observed under a laser scanning confocal microscope at different time intervals: 24 h, 36 h, 48 h, 72 h and 96-hpi. The exposed leaf was also observed at the same time intervals to assess protein secretion and accumulation of GFP signal in plant organelles. An analogous experiment was carried out using spores of the MoXYL1A-GFP strain rather than mycelia.

### Transient Expression of Proteins by Agroinfiltration on Tobacco Plants

Tobacco plants were grown in a chamber with conditions as follows: 8/16 h night/day at 22 °C. The effector protein genes *MoXYL1A/B* (amplified with the primer pairs pGDG-F/R given in Additional file 1: Table S1, using Guy11 cDNA as a template), were cloned into the pGDG vector and transformed into *Agrobacterium tumefaciens* GV3101 competent cells. The transformed bacterial strains were grown in LB media supplemented with antibiotics (200 µL/100 mL Rifampicin and 100 µL/100 mL Kanamycin) and incubated at 28 °C and 200 rpm in a shaking incubator. Independently, the rice chloroplast protein ChCPN10C (amplified with the primer pairs Os-CH-pGDR-F/R given in Additional file 1: Table S1, using rice protoplast cDNA as a template), was identified, and cloned into the pGDR vector and transformed in GV3101 competent cells following the same protocol. The pGDG and pGDR cultures at an OD_600_ of 0.5 were centrifuged at 5000 rpm for 5 min. The pellets were suspended in agroinfiltration buffer (prepared by mixing 10 mM MES, 10 mM MgCl_2_, and 150 μM acetosyringone in sterilized double distilled water). The pGDG and pGDR strains were combined and incubated at room temperature for 2–3-h. The strain suspension was inoculated on 6-week-old tobacco plants following the standard protocol of infection (Wang et al. 2011, 2017). The infected plants were kept in the dark for 48 h, and then the expression of GFP and RFP fluorescent proteins was observed under a confocal microscope at 488 nm and 561 nm wavelengths, respectively (Martin et al. 2009).

### Generation of Mutant and Complement Strains

The Split-marker approach was used to generate gene disruption mutants. Flanking regions 1.1 kb upstream (A fragment) and 1.1 kb downstream (B fragment) of both *MoXYL1A* and *MoXYL1B* were amplified and cloned in pCX62 vector to flank the hph cassette. The primer pairs MoXYL1A-AF/AR and MoXYL1A-BF/BR to amplify the A and B fragments and YG/F and HY/R for hph used with MoXYL1A-BR and MoXYL1A-AF, to obtain A-fragment/hygromycin, and B-fragment/hygromycin (BH and AH) fusion constructs, are given in Additional file 1: Table S1. The same approach was used for MoXYL1B using the respective primers provided in Additional file 1: Table S1. PEG-mediated fungal transformation was carried out using the Guy11 protoplast (Sweigard et al. 1992). The hygromycin-resistant transformants were screened with ORF and UAH primers (Additional file 1: Table S1) to identify candidate mutant strains. Southern blotting was performed to confirm gene replacement following the protocol given by (Norvienyeku et al. 2017). To generate double deletion mutants, the protocol given by Lin et al. (2019) was followed.

For complementation, 3 kb upstream and the full-length ORF, excluding the stop codons were amplified with the primer pair MoXYL1A and MoXYL1B-Comp-F and R (Additional file 1: Table S1) and cloned into pKNTG to generate GFP-fusion vectors. The GFP vectors were transformed into Guy11 protoplast and the protoplast of confirmed mutants and screened with ORF and UAH primers to identify the G418-resistant GFP-fusion and exact complements, respectively. GFP-fusion candidates were selected on the basis of PCR and GFP signal intensity.

### Infection Assays

One-week old Golden Promise Cultivar barley plants were used to conduct the virulence assay. The wild type strain Guy11, mutant strains (Δ*Moxyl1A* and Δ*Moxyl1B*), and the complemented strains were cultured in liquid CM, for 3-days at 28 °C in a 120 rpm shaking incubator. The isolates were drained, washed with sterile dd water, and media plugs were removed. Mycelia with moderate moisture were used to inoculate intact and abraded leaves of barley placed on moistened filter papers following established inoculation methods (Chen 2018) with slight modification. Incubation conditions were: 24 h in the dark, followed by 6-days in 12 h dark/12 h light at 28 °C. Disease severity was assessed, and photographs were taken on day 7 post-inoculation.

Two-week-old susceptible rice cultivar CO-39 was used to assess the disease development of mutants, wild type, and complemented strains. Strains were grown on RBM media for 7-days. Hyphae were scratched off, and plates were exposed to fluorescent light for 3-days to produce conidia. Conidia were then harvested from the plates and diluted with sterile double distilled water. Conidia count was conducted using a hemocytometer. An equal inoculum (based on conidium number) with 0.2% Tween-20 was prepared and sprayed on rice (Akagi et al. 2015). The inoculated rice plants were kept in a humid, dark growth room for the first 24 h. Later they were shifted in the light growth room. Disease phenotype was assessed at 7-days post-infection.

### Penetration Assays

Barley leaves were kept upside down in the moistened filter papers. Fungal spores were adjusted to the desired volume (5 × 10^4^ mL^−1^), with 0.2% Tween 20 and a 20 µL was used to inoculate leaves with the spores of each strain (Akagi et al. 2015). The inoculated barley plates were incubated following the conditions used in virulence trials (see above). The leaf sheath was peeled, and invasive hyphae were observed at 24 h, 36 h, 48 h, and 72 h under the optical microscope.

### Appressorium Formation Assays

20 μL conidial suspension (5 × 10^4^ mL^−1^) from wild type, mutants (Δ*Moxyl1A-3* and Δ*Moxyl1A-13*) and complemented strains were placed on hydrophobic Thermo Fisher Scientific coverslips to induce appressorium formation (Abdul et al. 2018; Aliyu et al. 2019). The incubation was done at 28 °C in a dark incubator and observed under a Nikon TiE system (Nikon, Japan) at 2 h, 4 h, 6 h, and 8 h, respectively.

### Conidiophoregenesis Assays

Asexual reproduction of the wild type, mutants, and complemented strains was assessed upon growth on RBM, SDC, and CM-II media. Plates were scratched on day 7 post-inoculation and kept in a light incubator at 28 °C. Conidiophore formation was observed at 12 h, 24 h, 36 h, and 48 h. To quantify spore production, conidia were washed off the plates and counted on a hemocytometer under a microscope.

### Cell Wall Stress Response

For cell wall sensitivity assays, CM media was supplemented with cell wall perturbing agents: sodium dodecyl sulphate (SDS, 0.01%), Calcofluor White (CFW, 200 μg/mL) or Congo Red (CR, 200 μg/mL) and cultured in the dark for 10-days at 28 °C. The colony diameter was measured on day 10 after inoculation. The inhibition rate was calculated as previously described (Zhang et al. 2014).

### Genomic DNA Extraction

For the Southern blot assay, total genomic DNA was extracted from the mutants, wild type and complemented strains grown in liquid CM shaken for 3-days at 120 rpm and 28 °C, using the SDS-CTAB DNA Extraction method (Aliyu et al. 2019). The resultant DNA suspension was then digested with Ste I Restriction enzyme for MoXYL1A and HindIII enzyme for MoXYL1B, and Southern blotting was performed as previously described (Norvienyeku et al. 2017).

### Real-Time RT-PCR Assay

Total RNA extraction was carried out following the previously described protocol (Lin et al. 2019). To check the expression of *MoXYL1A* and *MoXYL1B in-planta* and in individual deletion strains with quantitative real-time PCR (qRT-PCR), RNA extracted from the wild type strain Guy11 and the mutant strains (∆*Moxyl1A* and Δ*Moxyl1B)* were subjected to reverse transcription using SYBR® Premix Ex. Taq™ (TliRNaseH Plus). A reaction mixture of 25 μL was formulated using 12.5 μL of Premix Ex-Taq and 1 μL of each 10 μM primer (Additional file 1: Table S1) and 1 μL of cDNA template and incubated in the Eppendorf Realplex2 master cycler (Eppendorf AG 223341, Hamburg). Actin was used as positive control. The delta delta-CT method (2−^ΔΔCT^) was used for data analysis (Aliyu et al. 2019).

### Yeast-Two-Hybrid Assay

The pGBKT7 (AD) and pGBKT7 (BD) vectors were used for the construction of bait and prey constructs by In-fusion HD Cloning Kit (Clontech, USA). The CDS of respective genes were cloned and co-transformed into the AH109 yeast strain after sequencing. The Matchmaker Gal4 Two-Hybrid System 3 (Clontech, USA) was employed following the manufacturer’s guidelines. The positive transformants on SD-Trp-Leu medium were tested on SD-Trp-Leu-His-Ade medium, using the positive and negative controls from the Kit. The rich (YPD), lactate (YPL; 1% yeast extract, 2% peptone, 2% lactate), galactose (YPGal; 1% yeast extract, 2% peptone, 2% galactose), synthetic minimal with glucose (SMD; 0.67% yeast nitrogen base, 2% glucose, amino acids, and vitamins), synthetic minimal with lactate (SML; 0.67% yeast nitrogen base, 2% lactate, amino acids, and vitamins) or synthetic minimal with galactose (SMGal; 0.67% yeast nitrogen base, 2% galactose, amino acids, and vitamins) media were used for growth of yeast cells.

## Results

### Identification of *M. oryzae* Endo-1,4-Beta-Xylanase I and Generation of *∆Moxyl1* Strains

Domain-specific BLASTp search for the *Neurospora crassa* glycoside hydrolase family 11 domain amino acid sequence identified two GH11 family domain-containing proteins in *Magnaporthe oryzae* (*MoXYL1A*), encoded by *MGG_07955*, and *MoXYL1B* encoded by *MGG_08424*. To elucidate the physiological and pathological functions of *MoXYL1A* and *MoXYL1B* in *M. oryzae*, we generated targeted gene knock-out strains by replacing the coding region of *MoXYL1A* and *MoXYL1B* with the hygromycin phosphotransferase resistance (hph) gene using established homologous recombination techniques (Catlett et al. 2003). Putative *MoXYL1A* and *MoXYL1B* gene deletion transformants were selected on double layered TB3 agar containing 300 μg/mL (bottom layer) and 600 μg/mL (upper layer) hygromycin B and screened by PCR. Two successful knock-out strains each for *MoXYL1A (∆Moxyl1A-3* and *∆Moxyl1A-13*), and *MoXYL1B* (*∆Moxyl1B-5* and *∆Moxyl1B-7*) identified by PCR screening were checked using qRT-PCR and Southern Blotting (Additional file 1: Fig. S1). These assays confirmed the successful replacement of the *MoXYL1A* and *MoXYL1B* genes with *hph* in these strains (Additional file 1: Fig. S1). Our ability to recover deletion mutants indicates that survival of the rice blast fungus is independent of *MoXYL1A* and *MoXYL1B* function under standard conditions.

### Influence of *MoXYL1A *and *MoXYL1B* Gene Deletion on Vegetative and Asexual Growth of *M. oryzae*

**Fig. 1:**
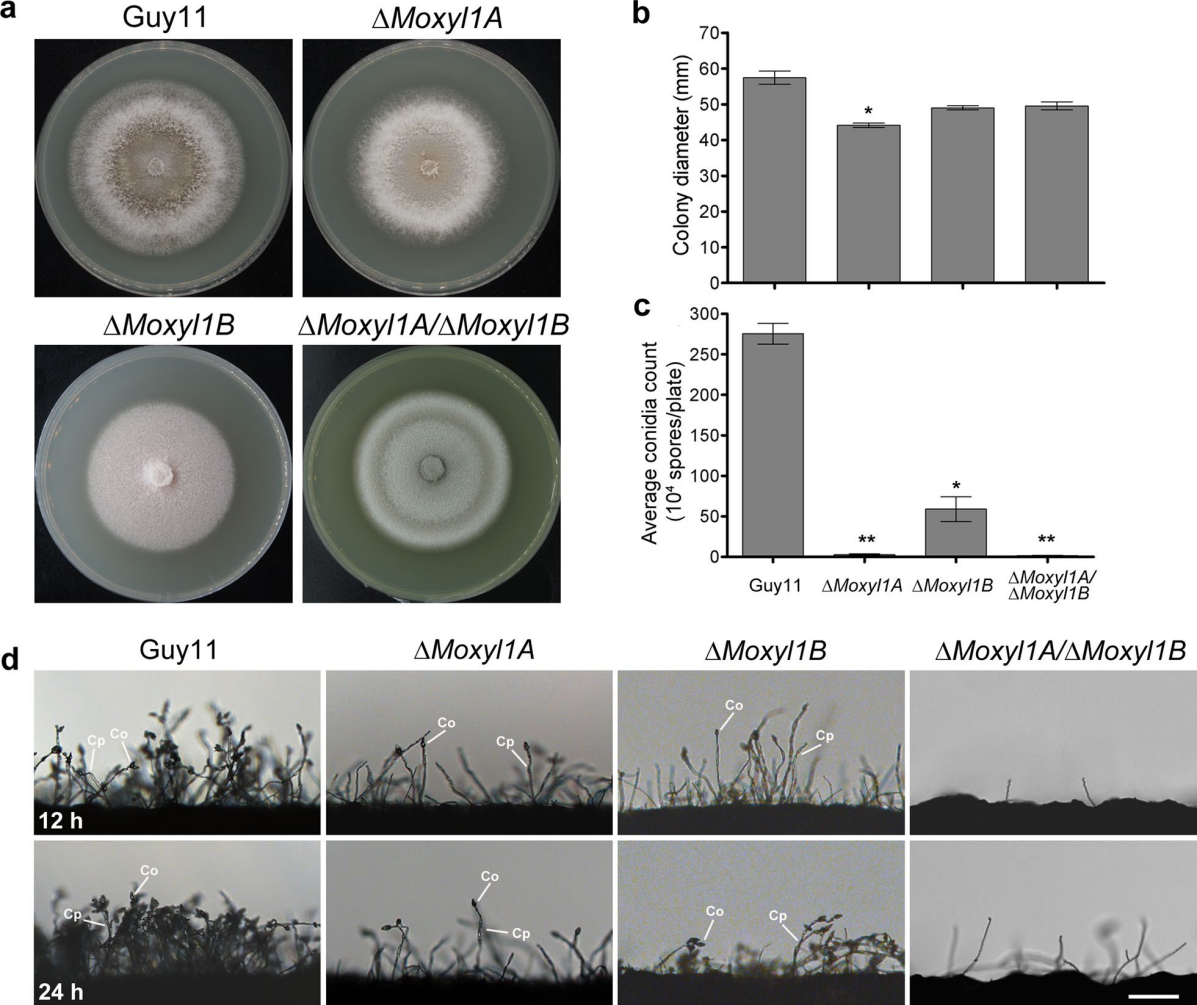
Impact of *MoXYL1* gene deletion on colony morphology and infectious growth of *M. oryzae*. **a** Strains of the indicated genotype were inoculated on CM media and photographed after 10-days of growth. **b** Statistical analysis of average colony diameter (mm) from three independent biological experiments with five replicates each. One-way ANOVA (non-parametric) was employed to assess statistical significance. Error bars account for standard deviation and asterisks represent the significant difference between wild type Guy11 and the mutant strain (*p* < 0.001). **c** Depicts drastically reduced ability of conidiophoregenesis of Δ*Moxyl1A* genes as compared to Guy11 at 12 h and 24 h interval. **d** Quantitation and statistical analysis of conidia production in Δ*Moxyl1* strains relative to Guy11, obtained from cultures grown on Rice Bran Media, Straw Decoction and Corn media and CM-II media, respectively, from five independent biological experiments with five replicates. The data was analyzed with GraphPad Prism5; error bars represent the standard deviation, while a single asterisk (*) represent significant differences (*p* < 0.05) and double asterisks (**) represent significant differences (*p* < 0.001) according to ordinary one-way ANOVA

To investigate the role of *MoXYL1* gene deletion on the growth of *M. oryzae*, mycelial plugs of single and double Δ*Moxyl1A* and Δ*Moxyl1B* mutants*,* wild type (Guy11) and the complemented mutant strains were inoculated on Complete Medium (CM) and incubated under dark conditions at 28℃ for 10-days. Growth measurements (mm) were taken on day 10 post-inoculation and plates were photographed. This assay showed no strong adverse effects on growth for all strains tested (Fig. 1a, b, Additional file 1: Fig. S2a, b). However, a noticeable reduction in aerial hyphae and minimal but statistically significant difference in colony diameter was observed for Δ*Moxyl1A* compared to WT. In contrast, there was no significant difference between Δ*Moxyl1B* and WT. The double deletion strain (DKO) was obtained via HR-based deletion of *MoXYL1B* on the Δ*Moxyl1A* background. Colony morphology and size of the double mutant was not significantly different from either single knockout. We conclude that *MoXYL1A* and *MoXYL1B* do not have specific morphogenesis-related functions in blast fungus under standard conditions.

A conidiophoregenesis assay was conducted to ascertain the impact of these mutants on asexual reproduction in *M. oryzae*, as conidiation plays a vital role in the survival and dissemination of the fungus (He et al. 2015). To quantify conidia production, conidia were harvested after 10-days, diluted with an optimized volume of sterile distilled water and then counted using a hemocytometer. The results showed that the Δ*Moxyl1A* and Δ*Moxyl1A*/Δ*Moxyl1B* strains were severely impaired in conidiophore production, with almost no conidia produced, while Δ*Moxyl1B* produced conidiophores of WT shape but in reduced number relative to WT (Fig. 1c). To further corroborate this defect, mutant and wild type strains were also grown on SDC and CM-II media and conidia were counted (Fig. 1d, Additional file 1: Fig. S2). The results confirmed a significant reduction in spore production in the deletion mutants, with a complete lack of conidiation in the double mutant, suggesting that there is clear contribution of these genes to the asexual development of *M. oryzae*, with *MoXYL1A* having an essential role and *MoXYL1B* a partial role in this growth phase. The conidiation defect of Δ*Moxyl1A* was partially rescued in the complemented strain; however, although it produced conidia that are morphologically indistinguishable from the wild type, the overall number was reduced. The defective conidiation was fully restored in the *MoXYL1B*-complemented strain (Additional file 1: Fig. S2).

### *MoXYL1A *is Required for Complete Virulence of *M. oryzae*

**Fig. 2:**
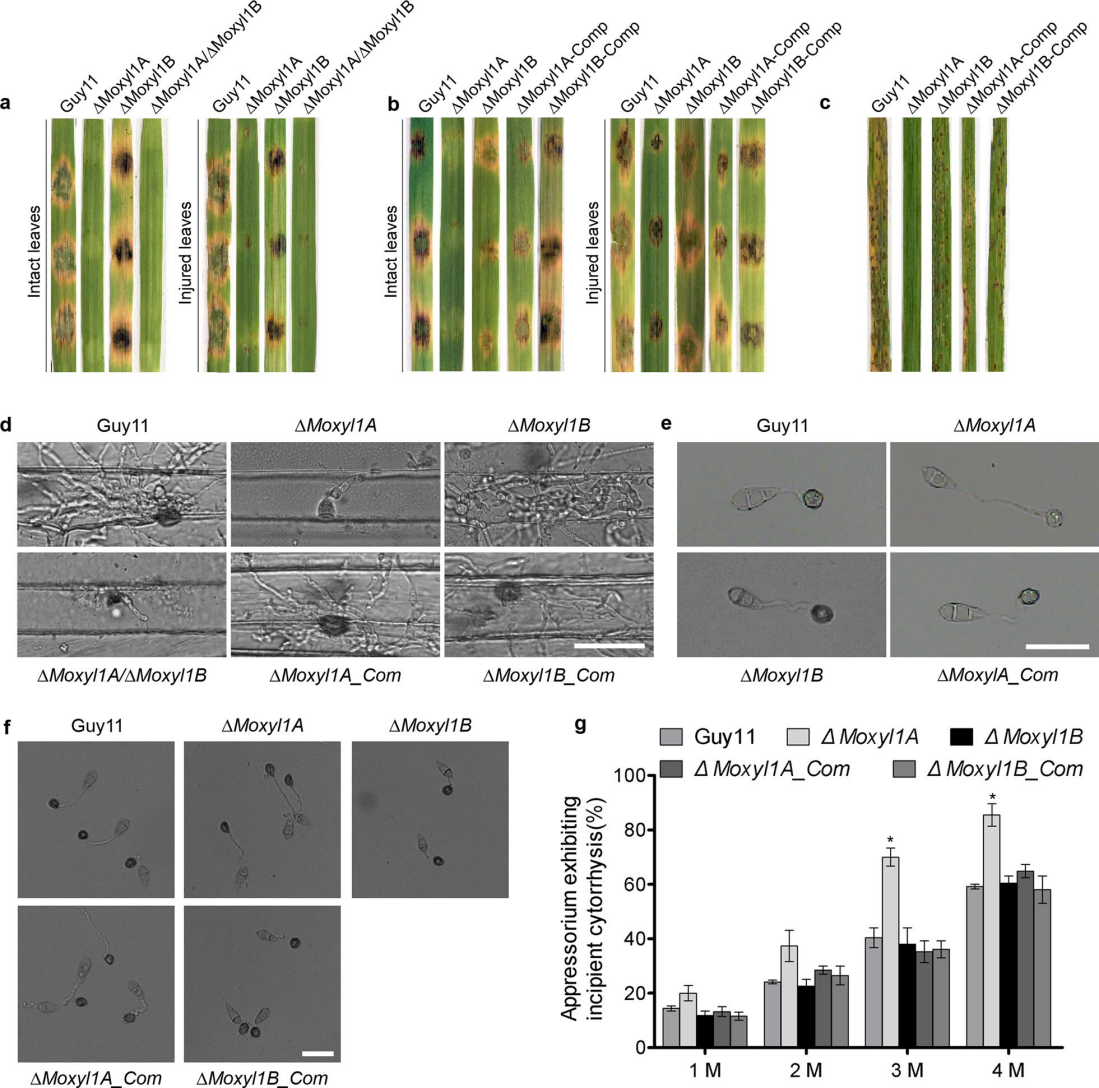
Targeted gene replacement of *MoXYL1A* compromised turgor-mediated appressorium integrity and impaired the virulence of *M. oryzae*. **a** Showed hyphae-mediated virulence characteristics of the individual strains on intact and injured leaves of one-week-old, barley seedlings.induction of blast lesion was assessed at 7-dpi. Images are representative of three independent assays, each assay with three replicates. **b** Virulence bioassay conducted on 21-days-old rice seedlings using spore-drop inoculation method. Conidial suspensions were prepared as 1 × 10^5^ conidia mL^−1^ in 0.2% Tween 20, for both the mutants and wild-type 20 µL. **c** Conidia-mediated pathogenicity/virulence characteristics Δ*Moxyl1A,* Δ*Moxyl1B,* and the wild-type on 21-days-old rice seedlings, through spray-inoculation with conidial suspensions (1 × 10^5^ conidia mL^−1^ in 0.2% Tween 20). **d** The micrograph portrays the inability of Δ*Moxyl1A* and double deletion mutants to invade and colonize barley tissue at 48hpi. The absence of invasive hyphae were evident in barley cells inoculated with Δ*Moxyl1A* or the double deletion strain. In contrast, numerous invasive hyphae were seen in the leaves inoculated with either Δ*Moxyl1B* or Guy11. Images are representative of n = 2 independent biological replicates. Scale bar, 20 μm. **e** Appressoria were produced artificially on Thermo-fisher hydrophobic coverslips and observed at 8 hpi. Images show non-functional appressoria lacking melanin lining for the Δ*Moxyl1A* mutant. **f** Showed results from incipient cytorrhysis assays performed to evaluate the turgidity of appressorium form by conidia from the Δ*Moxyl1A,* Δ*Moxyl1B,* Δ*Moxyl1A_Com.,* Δ*Moxyl1B_Com.,* and the wild-type hydrophobic coverslips for 8-h appressorium formed were treated with 2 M glycerol solutions. Collapse appressorium were counted using the Olympus DP80 light microscope. Scale bar = 20 μm. **g** The bar graph showed results from the statistical evaluation of the proportion of collapsed appressorium recorded in the Δ*Moxyl1A,* Δ*Moxyl1B,* Δ*Moxyl1A_Com.,* Δ*Moxyl1B_Com.,* and the wild-type strains on hydrophobic coverslips for 8-h and treated independently with varying concentrations (1 M, 2 M, 3 M, and 4 M) of glycerol solutions during incipient cytorrhysis assays. Consistent results from three independent biological experiments with each consisting of five technical replicates were used for statistical analyses. For each independent biological experiment, 100 appressoria were counted (n = 100*3). treatments yielding significant difference (*P* ≤ 0.05) are denoted with asterisks “*”

A susceptible rice cultivar CO39 and leaves detached from the Golden Promise cultivar of barley were used to conduct pathogenicity assays to assess the role of *MoXYL1* genes in the pathogenesis of rice blast fungus. These examinations revealed an impairment in the virulence characteristics displayed by the Δ*Moxyl1A* and Δ*Moxyl1A*/Δ*Moxyl1B* strains, which were unable to produce proficient blast lesions, while Δ*Moxyl1B* produced typical blast lesions (Fig. 2a). These results suggest *MoXYL1B* plays a dispensable role in pathogenicity while *MoXYL1A* plays a significant role in imparting virulence to *M. oryzae*. A comparable experiment was done using a spore suspension inoculated onto intact and abraded barley leaves. We infected with conidia of WT, single mutants, and complemented strains and observed similar pathogenicity defects for Δ*Moxyl1A* conidia as with mycelia. Virulence defects were rescued in the complemented strain (Fig. 2b). These results revealed the critical role of *MoXYL1A* in the pathogenicity of rice blast disease on barley.

We further conducted inoculation trials with spore suspensions (1 × 10^5^ conidia per mL in an aqueous solution of 0.2% Tween 20) on rice (cultivar CO39). Spore suspensions of wild-type and *MoXYL1A or MoXYL1B* mutant strains were independently and evenly sprayed on rice leaves. The inoculated seedlings were kept under proper incubation conditions (see Methods) for 7-days. This rice pathogenicity trial showed consistent results with the barley experiments, with Δ*Moxyl1A* and Δ*Moxyl1A*/Δ*Moxyl1B* strains completely lacking virulence compared to wild-type and the complemented strains (Fig. 2c).

To unravel the factors responsible for the impairment in pathogenicity of the *MoXYL1A* deletion mutants, we performed a penetration bioassay using barley as the host plant. We inoculated barley leaves obtained from one-week-old barley plants with conidia harvested from Δ*Moxyl1A* and wild type Guy11 to examine the penetration ability and colonization efficiency of the fungus. The results showed that the targeted gene replacement of *MoXYL1A* had a profound impact on the penetration and likely colonization abilities of *M. oryzae* as compared to wild-type. At 48hpi, for Δ*Moxyl1A*, no invasive hyphae were visualized inside the barley leaf when its sheath was excised and observed under the microscope, while wild-type micrographs showed pronounced invasive hyphae that were branched and colonizing adjacent cells. These results confirmed the inability of *MoXYL1A* mutants to invade host plants and cause blast disease. Consistent with earlier results, no penetration defects were observed for the *MoXYL1B* deletion (Fig. 2d).

To further investigate the reason for pathogenicity defect observed in the Δ*Moxyl1A* strain, we performed an appressorium formation assay to assess the efficiency of pathogenic dirrentiation in the Δ*Moxyl1A,* and Δ*Moxyl1B* strains compared to the wild-type and the complementation strain. The Δ*Moxyl1A* strain was unable to form a normal appressorium at 8 h of incubation on hydrophobic coverslips. The mutant produced an abnormal appressorium with a long germ tube and no melanin-ring, suggesting that it was a non-functional appressorium that could not penetrate and colonize the barley leaves (Fig. 2e). This phenotype was rescued by complementation of *MoXYL1A*. The *MoXYL1B* deletion mutant strains also had delayed appressorium formation but their appressoria were morphologically normal. As the double deletion mutants are unable to form conidia, we could not assess their appressorium development. Furthermore, we demonstrated that targeted replacement of *MoXYL1A* gene significantly attenuated the generation of turgor in the appressorium (Fig. 2f, g). These observations showed that MoXYL1A positively regulate appressorium integrity in the rice blast fungus possibly by modulating cellular parameters associated with the generation and accumulation turgor pressure.

### Δ***Moxyl1A*** and Δ***Moxyl1B*** are Sensitive to Cell Wall Stress

**Fig. 3:**
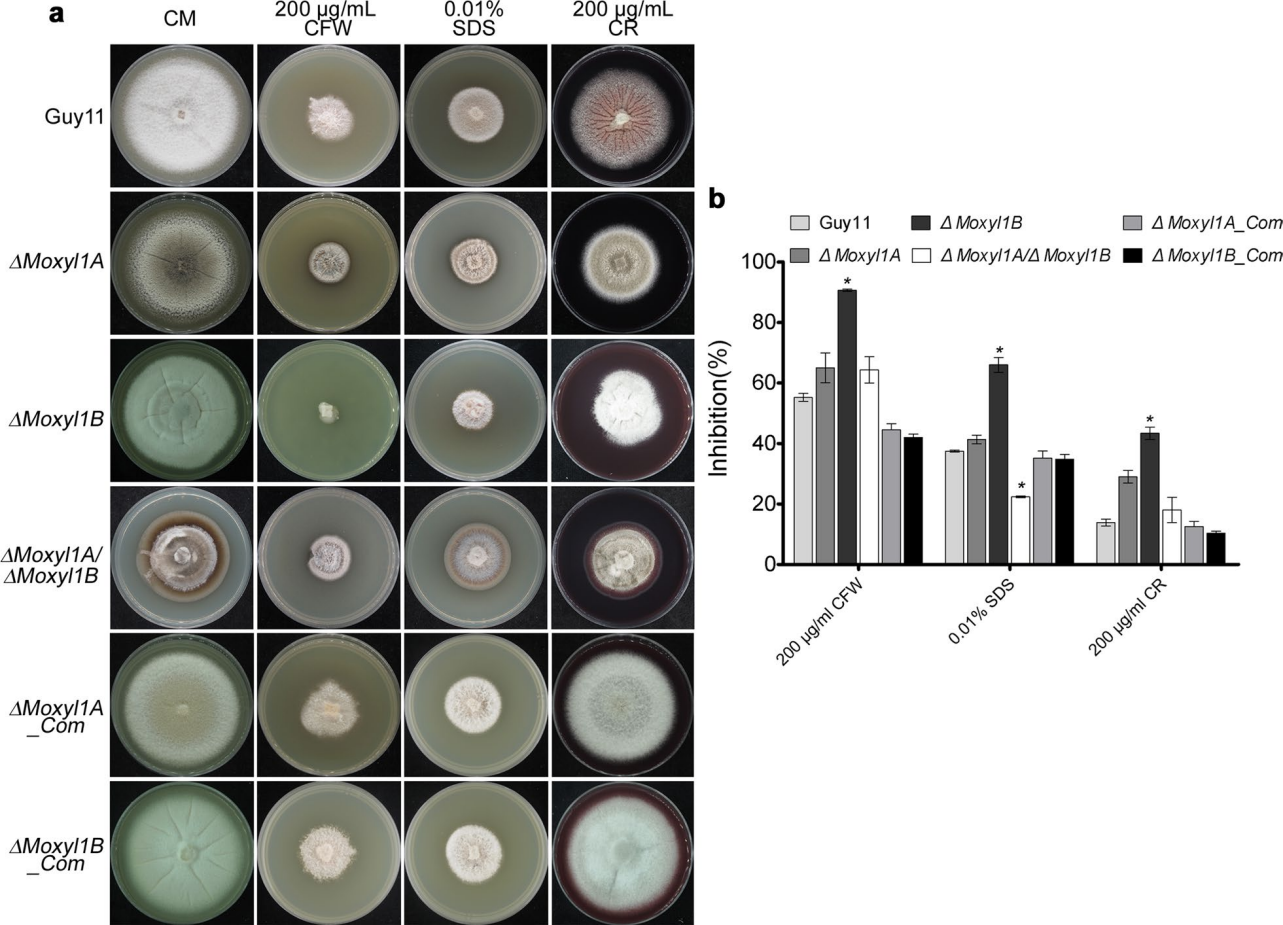
*MoXYL1A* and *MoXYL1B* mutants show varying degrees of sensitivity to cell wall perturbing agents. **a** Physical inhibitory effect of selected cell wall stress-inducing agents on the vegetative growth of the individual strains. The strain were cultured on CM media supplemented with 200 μg/mL Calcofluor White (CFW), 0.01% SDS or 200 μg/mL Congo Red (CR) for 10-days. **b** Quantification and statistical evaluation of the response of *MoXYL1* single and double deletion mutants and the wild-type strain to different cell wall stress inducing reagents. The inhibition data was generated from five independent biological experiments with five technical replicates each. One-way ANOVA (non-parametric) statistical analysis was carried out with GraphPad Prism8 and Microsoft Excel. Error bars represent standard deviation. Inhibition rate was calculated as a percentage = (the diameter of control − the diameter of treatment)/ (the diameter of control) × 100. Single asterisk represents a significant difference (*p* < 0.05)

Fungal cell wall integrity is crucial for infection of host cells, as the fungal cell wall maintains shape and facilitates exchange between the environment and fungus (Cabib et al. 2001). For proper growth and development, the cell wall requires repeated remodeling (Jeon et al. 2008). Therefore, we set out to assess the impact of cell wall-perturbing reagents on the growth of Δ*Moxyl1* strains*.* Calcofluor White (CFW) was used to test whether fungal strains are defective in cell wall assembly or have a defect in cell wall integrity (Lussier et al. 1997; Ram et al. 1994). Sodium dodecyl sulphate (SDS) is a detergent that compromises membrane stability, since cell wall defects increase the vulnerability of the plasma membrane to SDS, sensitivity can indicate problems with the cell wall (Bickle et al. 1998; Igual et al. 1996; Shimizu et al. 1994). CR, Congo Red (CR) is an additional cell wall stress reagent (Wood and Fulcher 1983). We supplemented CM culture media with Calcofluor White (200 µg/mL CFW), Congo Red (200 µg/mL CR), or sodium dodecyl sulphate (0.01% SDS) prior to inoculation with WT and mutant strains. Quantification of the growth inhibition rate, based on colony size, showed that the Δ*Moxyl1B* strain was more sensitive to cell wall stress reagents than Δ*Moxyl1A,* suggesting a possible role for this gene in cell wall integrity. Interestingly, we observed that double gene deletion, however, rescued the *MoXYL1B* phenotype to approximate that of the *MoXYL1A* single mutant, suggesting that the absence of *MoXYL1A* improves stress tolerance of Δ*Moxyl1B* strains (Fig. 3). From these observations, we speculated that MoXYL1A and MoXYL1B possibly modulates stress homeostasis in *M. oryzae* by counter regulating either expression, or enzymatic activities of each other.

### MoXYL1A and MoXYL1B Localize to the Cytoplasm in *M. oryzae*

**Fig. 4:**
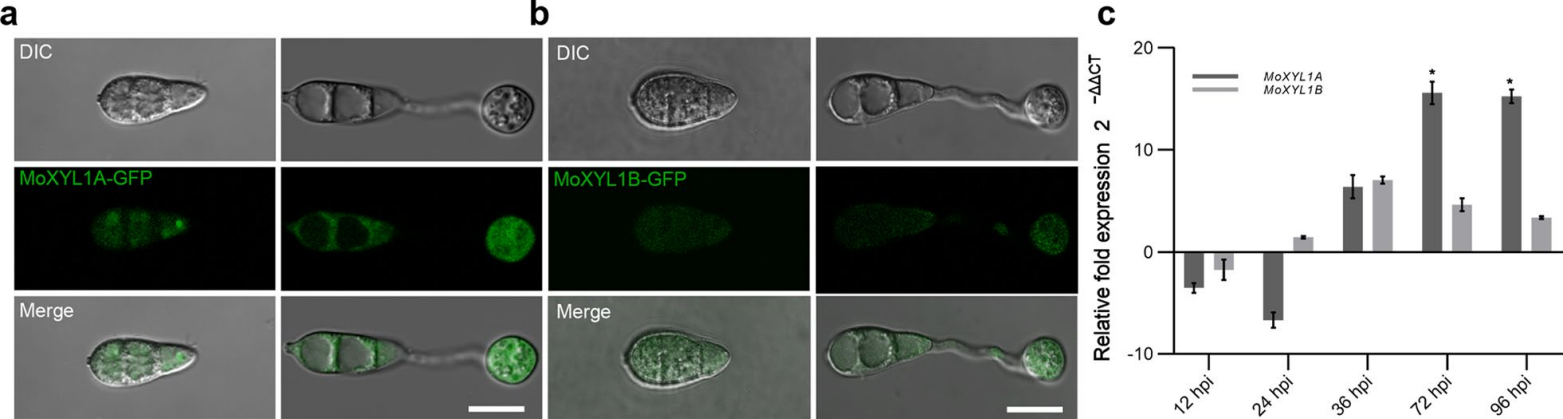
Subcellular localization of the relative expression xylanases at different stages of *M. oryzae -*host interaction. **a** Localization of MoXYL1A in the aberant conidia, conidia germination and appressorium formation stages of *M. oryzae* was determined by transforming a MoXYL1A-GFP fusion construct into the protoplast of the wild type strain Guy11 and examining fungal cells using the Scale bar = 20 µm. DIC indicates bright field illumination. GFP was excited at 488 nm. **b** Localization of MoXYL1B was assessed as in (**a**). MoXYL1B-GFP signal is evident in the conidium and appressorium. Scale bar = 20 µm. **c** In-planta expression of *MoXYL1A* and *MoXYL1B* transcripts during distinct stages of host–pathogen interaction was assessed by qRT-PCR. Vegetative hyphae were used as a control stage and the expression level of *MoXYL1A* and *MoXYL1B* at the hyphal stage was set to 1. Error bars represent standard deviation (SD). SD was calculated from three independent biological replicates along with three technical replicates. (*, *P* < 0.05 by t-test)

The subcellular localization of the MoXYL1A and MoXYL1B proteins in *M. oryzae* was investigated by transforming GFP fusion constructs of both proteins under their respective native promoters into the protoplast of the Guy11 strain (Dr. Didier Tharreau, CIRAD, Montpellier, France). The cultured strains harboring the florescence signals were observed with a Nikon laser confocal and laser excitation epifluorescence microscope, showing that both fusion proteins were mainly localized in the cytoplasm during vegetative and infectious development of the rice blast fungus (Fig. 4a, b). However, there was a weak GFP signal observed in conidia and the appressorium for MoXYL1B (Fig. 4b). To assess expression dynamics of these genes, the transcript levels of *MoXYL1A* and *MoXYL1B* were measured during host-plant interaction at varying intervals of infection. 6-week-old rice seedlings were infected with a spore suspension of WT *M. oryzae* and RNA was extracted from the infected plants at 12 h, 24 h, 36 h, 72 h and 96 h post inoculation for qRT-PCR assessment of *MoXYL1A* and *MoXYL1B*. Results showed that both *MoXYL1A* and *MoXYL1B* were not expressed at the hyphal stage, since control mycelia did not have detectable transcripts and we infer therefore that the genes are expressed below the limit of detection. In early infection stages, the expression of *MoXYL1A* and *MoXYL1B* was down-regulated, suggesting that these genes do not play any key role in initiation of the infection cycle (Fig. 4c). However, *MoXYL1A* expression was significantly upregulated at 72-hpi, suggesting that MoXYL1A has some regulatory role in the later infection stages of the disease cycle. The expression profile of *MoXYL1B* was not highly dynamic, suggesting that it is unlikely to play a major role in the infection process and may instead have some other regulatory roles in the fungus independent of pathogenicity.

### *M. oryzae* Likely Deploys MoXYL1A as Putative Cytoplasmic Effector Protein Targeting Host Chloroplast

**Fig. 5:**
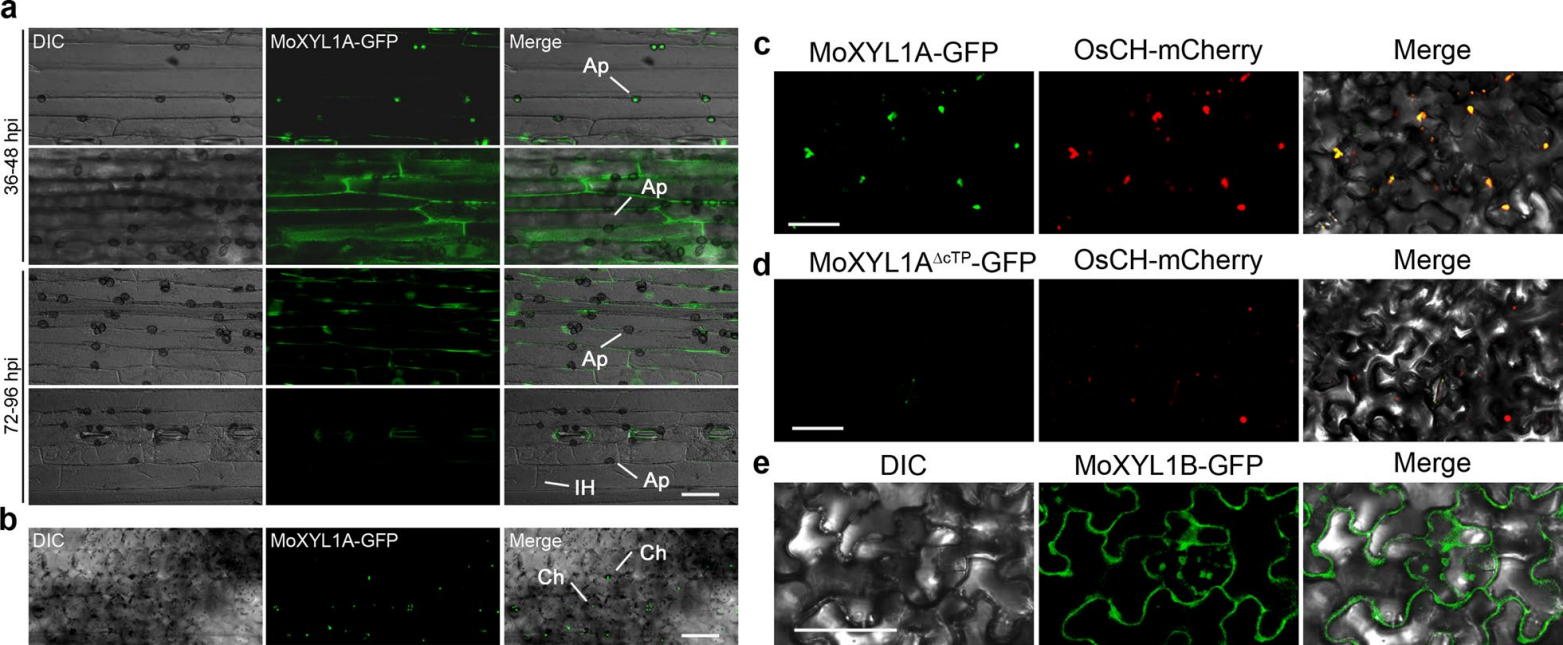
MoXYL1A accumulated at the Chloroplasts of barley and tobacco seedlings. **a** Showed the localization pattern of MoXYL1A-GFP during *M. oryzae* interaction with barley host during early (24–48-hpi), and late (72–96-hpi) stages of pathogen-host interaction. **b** The micrograph revealed the accumulation of MoXYL1A-GFP to the chloroplast of leaf epidermal tissues of barley leaves at 72-hpi. Scale bar = 10 µm. **c** and **d** The micrograph confirmed the co-expression of (MoXYL1A-GFP) and the chloroplast marker (ChCpn10) in the chloroplas of agro-infiltrated tobacco seedlings. Scale bar = 10 µm. **e** Showed distortions in the localization pattern of MoXYL1A-^∆ctp^-GFP. The MoXYL1A-^∆ctp^-GFP signals accumulated at the membrane or extracellular regions of agrobacterium infiltrated tobacco seedlings at 48-hpi. GFP was excited at 488 nm and RFP was excited at 561 nm. Scale bar = 20 µm

*Magnaporthe oryzae* mediates blast infection using appressorium-like structures produced on hyphal-tips (Kong et al. 2013). As noted earlier, the MoXYL1 genes were annotated as non-expressed xylanases in a prior study (Nguyen et al. 2011), which we posit was due to their potential secretion. To assess host localization of this effector protein, mycelial plugs from *M. oryzae* expressing MoXYL1A-GFP under its native promoter were used to inoculate barley plants and observed under a confocal microscope at different stages of disease development. Barley leaf sheath was peeled off to see the localization of the effector protein in host leaf cells. As fungal disease progressed through early stages, the invasive hyphae displayed GFP signal, and the effector protein was secreted out of hyphae at 72-hpi (Fig. 5a). At this time, the barley leaf was examined to track the translocation of effector proteins within the host, at which point it was trafficked to the chloroplast (Fig. 5b). The same chloroplast localization was observed upon inoculation with spore suspension.

Furthermore, we endeavored to verify the localization of MoXYL1A to rice chloroplasts. An *Agrobacterium tumefaciens*-based MoXYL1A-GFP construct driven by the CaMV35s promoter was generated and transiently co-expressed with the rice chloroplast marker protein ChCPN10C-RFP, in *Nicotiana benthamiana*. Using confocal microscopy to assess protein localization, at 48 hpi MoXYL1A-GFP and Ch-CPN10C-RFP were found to be co-localized in transfected tobacco cells, confirming the localization to the chloroplast of the effector protein (Fig. 5c). To ascertain the role of the chloroplast transit peptide in the chloroplast localization of the effector protein, we constructed GFP vectors with MoXYL1A lacking its chloroplast transit sequence (cTP) and co-expressed MoXYL1A-^Δctp^-GFP with Os-CH-RFP (a rice chloroplast marker protein) in tobacco plants. The deletion of the 42-amino acid cTP from MoXYL1A-GFP resulted in no observable GFP signal, confirming the requirement of the transit peptide for proper localization or targeting of MoXYL1A to the chloroplast (Fig. 5d). Consistent with results obtained from bioinformatic analyses, microscopy examination of the localization of MoXYL1B in leaves of tobacco seedlings transfected with *Agrobacterium* strains habouring the MoXYL1B-GFP constructs confirmed that MoXYL1B does not target any specific host organelle but instead accumulated at the perifery (extracellular region) of the host cells (Fig. 5e). We inferred that besides the promotion of vegetative growth, sporulation, and stress tolerance, MoXYL1A additionally functions as cytoplasmic effector protein that targets and possibly compromise the integrity of the chloroplast during pathogen-host interaction.

### The Impact of MoXYL1 Genes Deletion on the Expression of Pathogenicity-Related Genes During *M. oryzae*-Rice Interaction

**Fig. 6:**
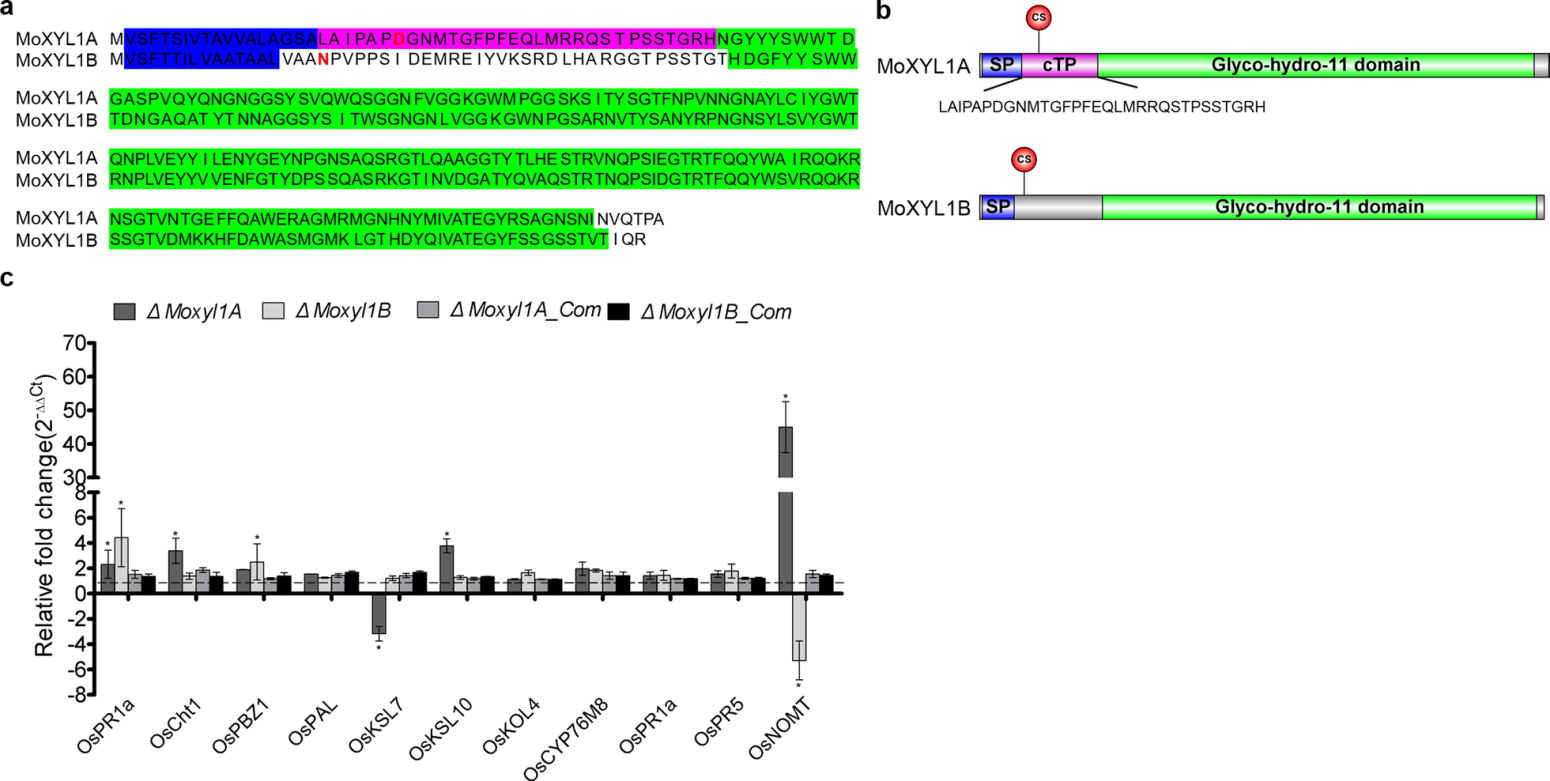
Comparative sequence features of *M. oryzae* XYL1s and expression dynamics for pathogenicity-related genes in rice seedlings inoculated with strains lacking MoXYL1s. **a** and **b** Comparative alignment results for MoXYL1A and MoXYL1B and detail sequence architecture for MoXYL1A and MoXYL1B. Sequences shaded in Blue denote the secretion signal peptides (SP), sequences shaded in Red denote the chloroplast targeting peptide (cTP), sequences shaded in Green denote the conserved Glyco-hydro_11 domain motif, and the cleavage site is denoted as (CS) **c** Showed the relative expression (in folds) of genes coding for pathogenicity-related proteins in rice blast susceptible CO39 cultivar inoculated with Δ*Moxyl1A,* Δ*Moxyl1B,* Δ*Moxyl1A_Com.,* Δ*Moxyl1B_Com.,* and the wild-type at 12-hpi. Error bars represent standard deviation (SD). SD was calculated from three independent biological replicates along with three technical replicates. (*, *P* < 0.05 by t-test)

Finally, comparative analyses of protein sequences of MoXYL1A and MoXYL1B with SignalP 5.0 (Teufel et al. 2022) confirmed both MoXYL1A and MoXYL1B posses the N-terminal secretion signal peptide. Meanwhile, TargetP-assisted analysis (Armenteros et al. 2019) identified a chloroplast targeting peptide (cTP) exclusively in MoXYL1A (Fig. 6a, b). From these observations, we posited that beyond secretion, *M. oryzae* deploys XYL1A as an effector to target and possibly compromise the defense capabilities of the chloroplast during pathogen-host interaction. Furthermore, we examined the expression pattern of pathogenicity-related genes, including Probenazole-inducible protein PBZ1/PR10B (Os12t0555200), pathogenesis-related protein1/PR1A (*Os07g0129200*), Thaumatin-like pathogenesis-related protein3 precursor/PR5 (*Os12g0628600*), KAURENE_SYNTHASE-LIKE_7/KSL7 (*Os02g0570400*), KAURENE_SYNTHASE-LIKE_10/KSL10 (*Os12g0491800*), syn-pimaradiene 3-monooxygenase/KOL4 (*Os06g0569500*), CytochromeP450/CYP76M8 (Os02g0569400), NARINGENIN_7-O-METHYLTRANSFERASE/NOMT (*Os12g0240900*), Phenylalanine ammonia-lyase/ (Os05g0427400), and chitinase1/Cht1 (*Os06g0726200*) (Jeon et al. 2020; Nie et al. 2019) in 14–21-days old CO39 rice seedlings independently challenged with the individual strains at 12-hpi. These examinations revealed a significant upregulation in the expression level of putative chloroplast localized PR genes (Additional file 1: Table S2), particularly, OsNOMT and OsKSL10 in rice seedlings inoculated with Δ*Moxyl1A* (Fig. 6c). We inferred that deploying MoXYL1s, especially MoXYL1A, possibly functions as a cytoplast effector that subverts host immunity by suppressing the expression of PR genes during pathogen-host interaction.

## Discussion

*MoXYL1A* and *MoXYL1B* belong to the glycosyl hydrolase family GH11 (Wu et al. 2006). The GH11 family is the pathogen-encoded GH group encoding xylanases with high substrate specificity (Paës et al. 2012). Many phytopathogenic fungi employ cell wall degrading enzymes to colonize their host (Annis and Goodwin 1997; Reignault et al. 2008; Have et al. 2002; Mary Wanjiru et al. 2002). However, not all genes encoding xylan-degrading enzymes play a role in the pathogenesis of the fungi that encode them (Gómez-Gómez et al. 2002; Wu et al. 1997). Given this discrepancy, we sought to characterize two *M. oryzae* cell wall degrading enzymes in the current work: Endo β-1,4-xylanases I *MoXYL1A* and *MoXYL1B*.

Barley plants infected with *M. oryzae* expressing MoXYL1A-GFP were used to determine if this effector protein is secreted into host cells. Transfer of GFP signal from invasive hyphae to plant cells was evident at 72-hpi. Given bioinformatic predictions, we further confirmed that the protein is released into host plant chloroplasts. In contrast, the related effector MoXYL1B-GFP did not traffic to host chloroplasts and was found to remain cytoplasmic. Plant chloroplasts act as integrators of disease and defense responses (Stael et al. 2015), yet very few effector proteins have been reported to target chloroplasts (Jelenska et al. 2010; Petre et al. 2016). As most parasitic microbes feed on host plant carbon compounds and thereby increase demand for photosynthesis, plant chloroplasts represent a crucial target of pathogens (Chen et al. 2010). In future, it will be of great interest to assess the role of MoXYL1A in host chloroplasts to better understand the pathogenesis mechanisms of blast fungus.

Previous transcriptomic profiling results revealed a substantial reduction in the expression patterns of MoXYL1A and MoXYL1B (Endo-β 1,4-xylanases) during early invasive growth *M. oryzae in-planta* (Nguyen et al. 2011). This study, we observed that MoXYL1A and MoXYL1B possess secretion signal peptide. Further transcriptomic analyses of the expression pattern of *MoXYL1A and MoXYL1B* at different stages of *M. oryzae-*host intaraction revealed about 3-folds increase in the expression pattern of *MoXYL1A* at 72-hpi (late stages of invasive growth), meanwhile, there was no visible changes in the expression of *MoXYL1B* during vegetative and invasive growth of the rice blast fungus. Also, we demonstrated that, while the deletion of *MoXYL1B* has no adverse effects on the pathogenicity or virulence attributes of the defective strains, the deletion of *MoXYL1A* severely compromised the virulence of the rice blast fungus. From these results, we speculated that the expression, particularly during late stages of infection and possibly the secretion of MoXYL1A is likely crucial in the pathogenesis *M. oryzae*.

Individual knockout of two endoxylanase I, particularly XYL1B genes has no obvious adverse effects on the vegetative growth of *M. oryzae*. *MoXYL1A* deletion had a mild negative effect on fungal growth, while *MoXYL1B* deletion had no effect. However, the sexual spores of this pathogen (conidia) are known to be a key determinant of fungal virulence, asexual spores are readily transported from sporulation sites (blast lesions) diseased plants nearby onto healthy host by wind current or water drops resulting in the rapid dissemination blast infection. The disease severity of blast fungus is therefore proportional to the number of conidia produced in blast lesions (Teng et al. 1991). Although *MoXYL1A* and *MoXYL1B* both have important conidiogenesis-related roles in rice blast fungus, with *MoXYL1A* particularly being indispensable for the asexual process, with mutant strains forming both fewer and deformed conidia across multiple conidiation-inducing media. Results from Yeast-two-Hybrid assays (Y2H) suggest the absence of physical interaction the two xylanases in *M. oryzae* (Additional file 1: Fig. S3) indicating that MoXYL1A and MoXYL1B influence sporogenesis in the rice blast fungus possibly by modulating independent pathways.

Successful penetration into and colonization of the host are two main factors contributing to the virulence of a fungal pathogen. For *M. oryzae*, penetration occurs within the first 24 h post-infection (Lim et al. 2018; Sun et al. 2017). The virulence of *MoXYL1A* mutants was severely compromised on both barley and rice plants, with mutant strains unable to penetrate the host cell. We speculated that the defects in pathogenicity of the mutant strains were caused by the inability to form a functional appressorium. Appressorium formation in *M. oryzae* is triggered by various stimuli emanating from both the environment and the host. The formation of functional appressoria is an essential infetion parameter in the disease cycle of rice blast fungus. Of the fewer conidia produced by *MoXYL1A* mutants, many (4/5) could not develop a normal appressorium. Also, the appressoria produced by these mutants failed to cause disease lesions on barley and rice plants. To further investigate the deficiency in appressorium formation, we used artificial induction of appressorium on hydrophobic coverslips and found that the mutant appressorium lacked the characteristic melanin layer. This layer is involved in cell wall assembly (Howard et al. 1991). The deposition of melanin is an essential cellular phenomenon that facilitates the generation of optimum turgor pressure required to support the formation of penetration peg used to breach the leaf cuticle and lead to fungal colonization (Chumley and Valent 1990; Howard and Valent 1996). We Demonstrated that the *MoXYL1A* mutant strains could not develop a proper host invasion machinery to enter and proliferate in the plant.

Fungal cell walls composed of a network of polysaccharides that play crucial roles in regulating the exchange of molecules between the cell and their environment (Jeon et al. 2008; Lipke and Ovalle 1998). Therefore, the sensitivity of *MoXYL1* defective strains to different types of cell-wall perturbing osmolytes indicated that both genes are vital for intact cell wall integrity. Also, the lack of melanin deposition in the cell wall of the *MoXYL1A* mutant strains likely accounted for the pronounce sensitivity of the defective strains to multiple stress-inducing agents. Also, the chloroplast contributes significantly to the enforcement of plant defense by facilitating the generation of diverse molecules, hormones, and proteins with antimicrobial or anti-parasitic properties (Kuźniak and Kopczewski 2020; Lu and Yao 2018). The observed up-regulations in the expression pattern of putative chloroplast destined PR genes, particularly in rice seedlings challenged with Δ*Moxyl1A,* suggest MoXYL1A, likely mitigates the survival of *M. oryzae in-planta* partly by suppressing the expression PR genes during pathogen-host interaction. Also, from the observed accumulation of MoXYL1B apparently to the plasma membrane or apoplastic region of *Agrobacterium* transfected leaf cells of tobacco seedlings, coupled with the absence of organelle targeting motif and the almost intact virulence characteristics recorded in the ∆*Moxyl1B* strains, we intimated that the functions of MoXYL1B as a secreted hydrolytic enzyme does not impact directly on the infection or virulence characteristics of *M. oryzae.* Pathogenic microbes met with hostile and stress endowed environments that threaten their survival and influence their ability to invade and colonize host tissues. Invading pathogens deploy a vast array of strategies to counter resistance posed by the potential host plants. Here, we showed that xylanases, particularly MoXYL1A, do not only contribute significantly to the stress tolerance of *M. oryzae* during physiological development but also support the survival of the rice blast fungus in-planta either targeting and subverting chloroplast integrity as a whole or partly by suppressing the expression of chloroplast associated PR genes during pathogen-host interaction. These observations underscore the potential significance of xylanases, especially MoXYL1A developing anti-blast strategies.

## Conclusion

In conclusion, we identified and cloned two endoxylanase-encoding genes, *MoXYL1A* and *MoXYL1B,* and found that endoxylanase I has a critical role in the asexual reproduction of the blast fungus *M. oryzae*. *MoXYL1A* but not *MoXYL1B*, is required for full virulence of the fungus. Deletion of endoxylanase I also compromises the cell wall integrity of *M. oryzae*. Moreover, the putative effector protein MoXYL1A is translocated to plant chloroplasts, though *MoXYL1B* does not target any plant organelle and instead accumulates in the plant cytoplasm. It is still unclear what the molecular role of *MoXYL1A* is in host chloroplasts and further insight into its roles in plant defense remain to be addressed. We further suggest that the chloroplast transit peptide sequence of *MoXYL1A* is important for the pathogenicity of rice blast fungus. We therefore propose that there might be some chloroplast protein essential for the effector to function appropriately in fungal virulence.

## Supplementary Information

**Additional file 1. Table S1**. List of primers used in this study.** Table S2**. Predicted location of pathogenicityrelated protein in rice.** Figure S1**. Single copy insertion confirmed by Southern Blot.** Figure S2**. Complementation of MoXYL1A and MoXYLB rescued the defects exhibited by the mutant strains.** Figures S3**. Y2H-mediated interaction pattern between putative* M. oryzae* xylanases A1 and 1B.

## Data Availability

The data that support the findings of this study are available from the corresponding author upon reasonable request.

## Abbreviations

CM: Complete Medium
CR: Congo Red
CW: Calcofluor White
CWDEs: Cell wall degrading enzymes
GHs: Glycoside hydrolases
GH10: Glycoside hydrolase 10
GH11: Glycoside hydrolase 11
HPH: Hygromycin phosphotransferase
MM: Minimum media
DKO: Double deletion strain
WT: Wild-type
ANOVA: Analysis of variance
CFW: Calcofluor white
SDS: Sodium dodecyl sulphate
GFP: Green fluorescent protein
SD: Standard deviation
qRT-PCR: Quantitative real-time polymerase chain reaction
cTP: Chloroplast transit peptide
RFP: Red fluorescent protein
Y2H: Yeast-two-hybrid assays
RBM: Rice Bran Media
SDC: Straw Decoction and Corn Media
LB: Lysogeny Broth
BH and AH: A-fragment/hygromycin, and B-fragment/hygromycin
ORF: Open reading frame
UAH: Upstream sequance fused to hygromycin
SMD: Synthetic minimal with glucose
SML: Synthetic minimal with lactate
SMGal: Synthetic minimal with galactose
PR: Pathogenicity-related
