# Supplementary material for: Magnaporthe oryzae Chloroplast Targeting Endo-β-1,4-Xylanase I MoXYL1A Regulates Conidiation, Appressorium Maturation and Virulence of the Rice Blast Fungus
Source: Rice (N Y). 2022 Aug 12;15:44. doi: 10.1186/s12284-022-00584-2 (PMC9374862; doi:10.1186/s12284-022-00584-2)
Supplement: Supplementary file 1 — Additional file 1. Table S1. List of primers used in this study. Table S2. Predicted location of pathogenicityrelated protein in rice. Figure S1. Single copy insertion confirmed by Southern Blot. Figure S2. Complementation of MoXYL1A and MoXYLB rescued the defects exhibited by the mutant strains. Figures S3. Y2H-mediated interaction pattern between putative M. oryzae xylanases A1 and 1B. [file 12284_2022_584_MOESM1_ESM.pdf]

Additional information

Table S1. List of primers used in this study

Primer Name	Sequence
MoXYL1A-AF	5' CGACCGAACCAGGATTTGC 3'
MoXYL1A-AR	5' GAAGATGTTTGAACCGCACC 3'
MoXYL1A-BF	5' CGGCGAGTTGATGTTGA 3'
MoXYL1A-BR	5' CAAGGGCATAGAAGCGAAT 3'
MoXYL1A-OF	5’ GCAGCTCATGCGGCGACAGT 3'
MoXYL1A-OR	5’ CCCTCGGTGGCGACAATC 3’
MoXYL1A-UF	5’ CACTTGCGTTAGCCTGAG 3'
MoXYL1A-UR	5’ CACAGTTTGCCAGTGATACA 3'
MoXYL1A-COMP-F	5'AACATCAGGCAGCTCGTA 3'
MoXYL1A-COMP-R	5'CGCCGGAGTCTGAACGTTGA 3'
MoXYL1A-qPCR-F	5' ATGGCAACATGACGGGATTC 3'
MoXYL1A-qPCR-R	5' ACTGCCACTGAACGCTGTAG 3'
MoXYL1A-pGDG-F	5' ATGGTCTCCTTCACCTCCATC 3'
MoXYL1A-pGDG-R	5' TCACGCCGGAGTCTGAA 3'
MoXYL1A-Y2H-F	5' ATGGTCTCCTTCACCTCCATC 3'
MoXYL1A-Y2H-R	5' TCACGCCGGAGTCTGAA 3'
MoXYL1B-AF	5’ ACGAGGTGATTTACTTTGGG 3'
MoXYL1B-AR	5’ TCTGGCTCTATGATGTATGTCTT 3'
MoXYL1B-BF	5’ GCGAGATCGACTGTAACAAACC 3'
MoXYL1B-BR	5’ ACGGAGGAAAGTGACCTACCATT 3'
MoXYL1B-OF	5’ GGGGAGTGCTCGGTATGTTT 3'
MoXYL1B-OR	5’ GGCGACGATCTGGTAGTCGT 3'
MoXYL1B-UF	5’ TTCAGACAAGACGCAACCC 3'
MoXYL1B-UR	5’ GCTCCATACAAGCCAACCAC 3'
MoXYL1B-COMP-F	5'AACATCAGGCAGCTCGTA 3'
MoXYL1B-COMP-R	5'CGCCGGAGTCTGAACGTTGA 3'

MoXYL1B-qPCR-F	5' ATGGCAACATGACGGGATTC 3'
MoXYL1B-qPCR-R	5' ACTGCCACTGAACGCTGTAG 3'
MoXYL1B-pGDG-F	5' ATGGTCTCCTTCACCAC 3'
MoXYL1B-pGDG-R	5' TCACCGCTGGATGGTGA 3'
MoXYL1B-Y2H-F	5' ATGGTCTCCTTCACCAC 3'
MoXYL1B-Y2H-R	5' TCACCGCTGGATGGTGA 3'
Os-CH-pGDR-F	5' ATGTCGTCGGTGCAGCT 3'
Os-CH-pGDR-R	5' TCAAGATAGGACAGCCATCA 3'
G418-F2	5' CAACAACACGCATCATCCCA 3'
G418-R2	5' TCAGAAGAACTCGTCAAGAA 3'
rice-actin-F	5' GCGTGGACAAAGTTTTCAACCG 3'
rice-actin-R	5' TCTGGTACCCTCATCAGGCATC 3'
OsPR1a-RT-F	5' GGAAGTACGGCGAGAACATC 3'
OsPR1a-RT-R	5' GGCGAGTAGTTGCAGGTGAT 3'
OsCht1-RT-F	5' CGTGGTGACCAACATCATCA 3'
OsCht1-RT-R	5' GAGTTGAAAGGCCTCTGGTTGT 3'
OsPBZ1-F	5' CTGTGGAAGGTCTGCTTGGA 3'
OsPBZ1-R	5' TCTTGTATACGCTCCCTGCG 3'
OsPAL-F	5' GAGATCAACTCCGTCAAC 3'
OsPAL-R	5' TGTAGAAGTCGTTCACCA 3'
OsPR1a-F	5' GTCGGAGAAGCAGTGGTA 3'
OsPR1a-R	5' CGAGTAGTTGCAGGTGATG 3'
OsPR5-F	5' CAGCCAGGACTTCTACGA 3'
OsPR5-R	5' TGTGTCTTGGTGTTGTCTTC 3'
OsKSL7-F	5' CCATATCCACAGCCAACA 3'
OsKSL7-R	5' ACTTAGTCCTCTCCTGATGT 3'
OsKSL10-F	5' TAACCCTTGCCTCTGGGATG 3'
OsKSL10-R	5' ATTGTTCCACCGGAATCCCT 3'
OsKOL4-F	5' GTGCACAGCTGACAGATGAC 3'

OsKOL4-R	5' TCGGATCTCTTGGTAGAGTAGC 3'
OsCYP76M8-F	5' ATGGAGAATAGCCAGATGTG 3'
OsCYP76M8-R	5' AAGTGTAGGTTGCCGATG 3'
OsNOMT-F	5' GAAGGTGTGGAGCATATCGG 3'
OsNOMT-R	5' GTGCAGAATCCACTTGAGC 3'

Table S2. Predicterd location of pathogenicity-related protein in rice.

Pathogenicity-related genes in rice	Abbreviated annotation	Gene accession number (Gene ID)	Predicted localization
Probenazole-inducible protein	OsPBZ1	Os12g0555200	Extracellular, Mitochondrial, ER, Peroxisomal, Chloroplast
Pathogenesis-related protein 1a	OsPR1a	Os07g0129200-01	Extracellular, and Mitochondrial
Thaumatin-like pathogenesis-related protein 3 precursor	OsPR5	Os12g0628600	Vacuolar, and Extracellular
ent kaurene synthase 7	OsKSL7	Os02g0570400	Extracellular, and Vacuolar
kaurene synthase like 10	OsKSL10	Os12g0491800	Cytoplasmic, Mitochondrial, and Chloroplast
Ent-kaurene oxidase 4	OsKOL4	Os06g0569500	Mitochondrial, Peroxisomal, and Chloroplast
Cytochrome P450 family protein	OsCYP76M8	Os02g0569400	Plasma membrane, Mitochondrial, and ER
naringenin 7-O-methyltransferase	OsNOMT	Os12g0240900	Extracellular, Mitochondrial, ER, Peroxisomal, and Chloroplast
Chitinase 1	OsCHT1	Os06g0726200	Extracellular, Vacuolar

phenylalanine ammonia-lyase	OsPAL	Os05g0427400	Cytoplasmic, Chloroplast

Additional figures

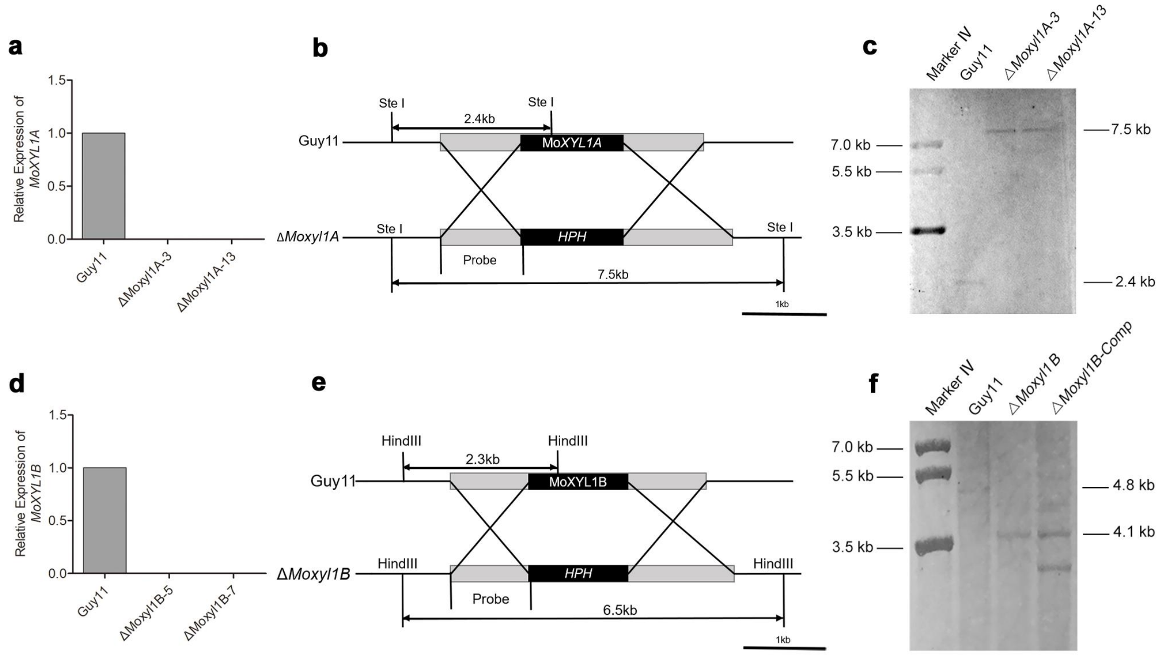

Figure S1. Single copy insertions confirmed by Southern Blot and relative expression of *MoXYL1A* gene in Guy11 and the two putative mutant strains was assessed by qRT-PCR. (a) Schematic of probe design for Southern blot for *MoXYL1A* gene. (b) Successful gene replacement of *MoXYL1A* by single insertion of *hph* at the *MoXYL1A* locus by Southern blot analysis. (c) The relative expression of *XYL1B* gene in Guy11 and the putative mutant strains was assessed by qRT-PCR. (d) Schematic of probe design for Southern blot for *MoXYL1B* gene (e) Successful gene replacement of *MoXYL1B* by single insertion of *hph* at the *MoXYL1B* locus by Southern blot analysis.

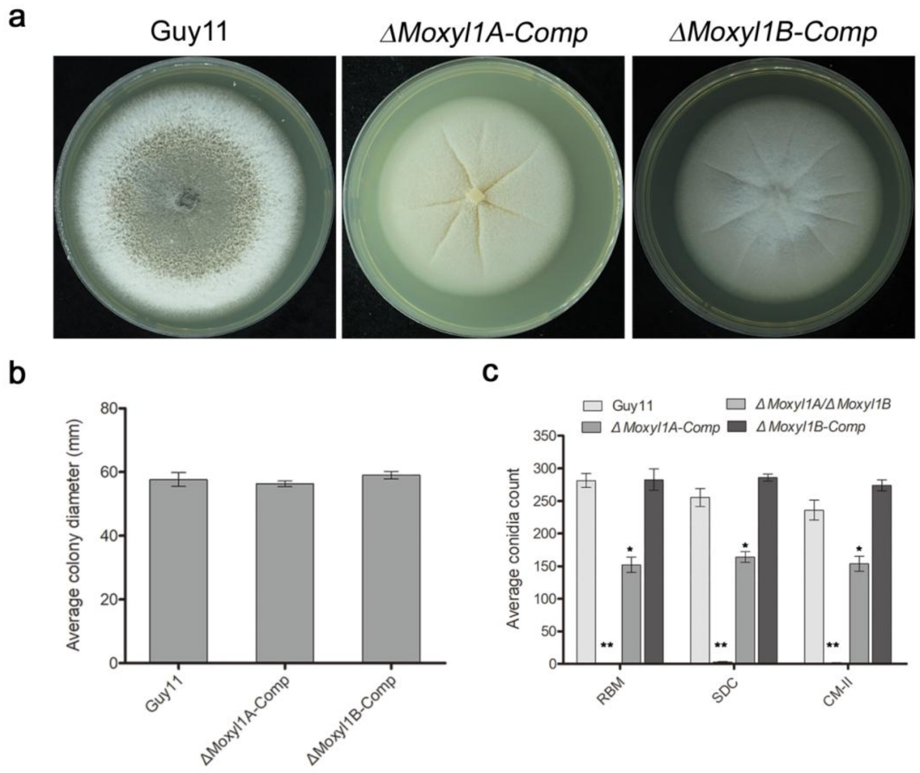

Figure S2. Complementation of MoXYL1A and MoXYL1B rescued the defects exhibited by the mutant strains (a) Photographs showing colony growth diameter (mm) of complemented deletion strains for *MoXYL1A&B* compared to WT. (b-c) Quantification of colony growth in (a) Quantification of conidiation in the complemented strains and the double mutant.

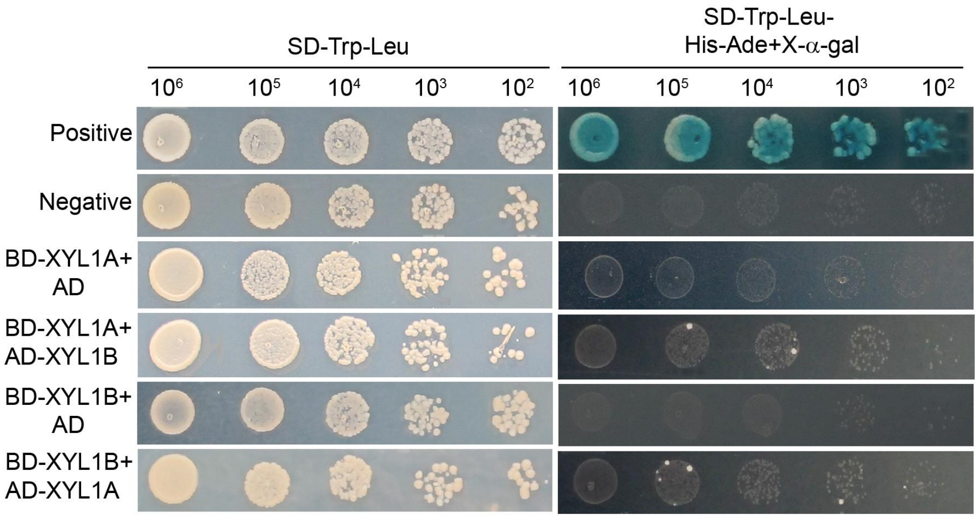

Figure S3. Interaction in Y2H and in-planta expression of putative *M. oryzae* xylanases 1A and B

(a) Yeast-2-hybrid experiments to assess interaction between the xylanases 1A and B in *M. oryzae* did not show physical interaction between the two proteins.
